# Real-time clinical analytics at scale: a platform built on large language models-powered knowledge graphs

**DOI:** 10.1093/jamiaopen/ooaf167

**Published:** 2026-01-06

**Authors:** Shuang Cao, Rui Li, Rui Wu, Ruihua Liu, Alexandre Duprey, Jason Zhao

**Affiliations:** Department of AI, Hill Research, Princeton, NJ 08540, United States; Department of AI, Hill Research, Princeton, NJ 08540, United States; Department of AI, Hill Research, Princeton, NJ 08540, United States; Department of AI, Hill Research, Princeton, NJ 08540, United States; Department of AI, Hill Research, Princeton, NJ 08540, United States; Department of AI, Hill Research, Princeton, NJ 08540, United States

**Keywords:** knowledge bases, clinical trials as topic, natural language processing, machine learning, information storage and retrieval

## Abstract

**Objectives:**

The increasing volume of clinical trial documents presents a significant challenge for biomedical researchers who must analyze vast amounts of unstructured and structured data. Traditional methods are no longer feasible given the scale and complexity of modern clinical trials.

**Materials and Methods:**

We introduce a large-scale clinical analytics platform, ClinicalMind, that integrates Large Language Models (LLMs) with Knowledge Graph technology to perform real-time clinical analytics over 110 000 clinical documents and 60 000 electronic medical records. The system employs a 2-phase graph update strategy and hardware acceleration to increase the accuracy and speed.

**Results:**

Our platform achieves an average query delay of 1.7 seconds with high accuracy (BLEU score: 0.85, ROUGE score: 0.92). The system can process and analyze thousands of clinical documents in real-time, significantly outperforming existing methods.

**Discussion:**

These results demonstrate that combining LLMs with a continuously updated knowledge graph enables scalable, low-latency clinical analytics across large and heterogeneous data sources. The observed performance gains highlight the potential of this approach to support real-time clinical decision-making and large-scale evidence synthesis, addressing key limitations of existing document-centric and retrieval-based methods.

**Conclusion:**

We demonstrate that our platform offers an efficient, scalable solution for real-time clinical analytics, enabling rapid analysis of large-scale clinical document collections.

## Introduction

The analysis of clinical trial documents is a critical task for biomedical researchers, offering insights into drug efficacy, patient outcomes, and treatment guidelines. However, with the explosion of clinical data generated by medical institutions, pharmaceutical companies, and research organizations, bioscientists face significant challenges in processing, integrating, and analyzing this vast amount of data.^1^^,^^2^ Traditional methods, such as manual curation and review, are no longer feasible due to the sheer scale of information, which may involve thousands of documents from different trials, each containing diverse and often unstructured data.

Recent advances in natural language processing (NLP) and machine learning have led to the development of automation technologies to assist in the analysis of large-scale clinical documents. Large Language Models (LLMs) and Retrieval-Augmented Generation (RAG) frameworks, which combine document retrieval with generation-based models, have been proposed as solutions to automate the understanding of clinical texts. GraphRAG,^3^ a technology that builds a Knowledge Graph (KG) from retrieved documents for enhanced reasoning, further extends RAG by representing clinical entities and their relationships in a structured format that can be traversed and queried.

Despite these advancements, existing methods still struggle to scale effectively when performing cross-document analysis across hundreds of thousands of clinical trials. One key limitation is that current LLM and RAG-based approaches focus primarily on single-document or small-scale multi-document tasks, which lack the ability to conduct comprehensive, cross-document knowledge synthesis at massive scales and within tight computational time constraints. This bottleneck becomes particularly critical when bioscientists need to perform real-time analysis for decision-making, such as identifying new drug effects or off-label uses across multiple clinical trials.

To address these challenges, we introduce a large-scale clinical analytic platform which is initialized from 300 curated sources and subsequently expanded with updates from 110 000 clinical documents and 60 000 electronic medical records (EMRs). Our system leverages the LLM and KG techniques to integrate both structured data (eg, EMRs) and unstructured text (eg, clinical trial papers), automatically discovering graph topologies and continuously updating the KG as new data is ingested. We employ hardware acceleration and parallel data pipelines to ensure that the system can handle large-scale document processing, graph traversal, and real-time query execution. Through extensive evaluation, we demonstrate that our system can provide answers to user’s clinical questions in 1.7 seconds with high accuracy. These results outperform existing methods, particularly in scenarios requiring large-scale, cross-document analysis.

## Background

A KG for clinical trials serves as a structured abstraction that represents clinical entities, such as drugs, diseases, treatments, and outcomes, and the relationships among them. This section provides technical details on the abstraction, initialization, updates, querying, and storage of the KG, specifically for clinical trial domain requirements.

### Abstraction for a clinical KG

There are 3 core concepts. Entities (Nodes) represent key components of clinical trials, including drugs, diseases, patient outcomes, clinical trials, side effects, etc Relationships (Edges) define connections between entities, including *Treats* which connects a drug to the disease it treats, *Causes* which connects a drug to a side effect, *Results In* which connects a clinical trial to a patient outcome, *Associated With* which links diseases to common symptoms or risk factors, etc Properties are associated with entities and relationships. For instance, clinical trials can have start dates, end dates, and trial phases, which are the associated properties.

### KG initialization

The KG is initialized by extracting entities and relationships from predefined sources like medical textbooks, official clinical trial guidelines, and structured clinical datasets.

The system follows these steps: (1) Entity Recognition and Extraction: Named Entity Recognition (NER) models are commonly used to identify and extract clinical entities like medications, conditions, and clinical outcomes from unstructured data sources (eg, medical literature and clinical guidelines). Popular NER models include BioBERT^4^ and ClinicalBERT.^5^ (2) Relation Extraction (RE): Relationships between entities are identified using relation extraction techniques, which process natural language descriptions to infer logical connections such as “drug X treats disease Y” or “drug X causes side effect Z.”^6^^,^^7^ (3) Graph Initialization: Once entities and relationships are extracted, they are added to the KG as nodes and edges, respectively, forming the initial graph structure. At this stage, domain-specific ontologies, such as SNOMED CT^8^ or UMLS,^9^ are used to standardize medical terms and relationships. These ontologies ensure that extracted entities (eg, drugs, diseases, outcomes) and their connections are consistently represented across the KG, even when different terminologies are used in the original data sources. This step is crucial for creating a unified and coherent graph that can integrate heterogeneous medical data from various sources.

### Storing the KG

The KG is stored in a graph database Neo4j.^10^ It allows efficient storage, querying, and traversal of graph structures. The KG is stored in the following format:

1. Nodes: Each node in the graph represents an entity (eg, drug, disease, outcome). Nodes have properties, such as names, identifiers, and metadata.
2. Edges: Each edge represents a relationship between 2 entities (eg, “treats,” “causes”). Edges can have properties such as confidence scores, references, or timestamps.
3. Indices: To optimize query performance, indices are created on frequently queried attributes (eg, drug names, disease types).

### Updating the KG

The KG is updated dynamically by parsing new clinical trial documents and structured clinical data (eg, EMRs) as they become available.

The system supports both batch updates, where large datasets are ingested periodically, and real-time updates, where new data is continuously ingested as it arrives. Batch updates involve parsing newly published clinical trial results or updated medical guidelines at regular intervals. During this process, new entities and relationships are added to the KG, while existing entities are updated if new information is identified, such as the discovery of a new side effect for a drug. In contrast, real-time updates occur as new clinical data, such as EMR entries, are received. The system processes this data immediately, ensuring the KG reflects the latest findings or modifications to existing entities and relationships.

### Querying the KG

Once the KG is constructed and updated, users can query the KG to retrieve information. Queries can range from simple entity retrievals (eg, “What drugs are used to treat lung cancer?”) to complex path-based queries (eg, “What are the survival rates associated with Pembrolizumab in treating lung cancer?”).

The queries are executed using graph query language Cypher.^11^ The system supports various types of queries, including entity retrieval, path queries, and subgraph queries. Entity retrieval allows the system to retrieve entities based on specific attributes. In the medical context, this could involve querying the system to find all drugs used to treat a particular disease, such as asking “What drugs are used to treat diabetes?” Path queries focus on discovering relationships between entities by traversing the graph. For instance, a query might aim to find the chain of relationships between a drug and its side effects through intermediate clinical trials, such as “Drug A was used in Trial X, which reported Side Effect B.” This process explores connections between nodes via intermediate edges. Subgraph queries retrieve a relevant portion of the LG that can address broader questions or support machine learning models. For example, users could request all data related to a specific clinical trial, including information about the drugs tested, patient outcomes, and side effects. The returned subgraph could then be used for further analysis, summarization, or report generation by an LLM.

## Methods

In this section, we discuss the design considerations and optimizations of ClinicalMind to construct clinical KG for real-time, large-scale clinical analysis.

### KG topology discovery

Generating an initial topology for a clinical KG is a crucial and time-consuming task, as the domain knowledge spans diverse medical concepts such as diseases, drugs, treatments, and clinical outcomes. We leverage selected authoritative medical guidelines and textbooks to construct most of the KG’s nodes at the topology discovery stage, significantly reducing the cost and frequency of LLM invocations during later KG updates.

Our selection process from authoritative medical sources was guided by clinical relevance and evidence quality. We prioritized concepts that directly influence treatment decisions, such as drug-disease interactions with established efficacy data.^12^^,^^13^ Entities with extensive documented relationships to other clinical concepts were favored to ensure robust graph connectivity.^14^^,^^15^ Additionally, we limited inclusion to concepts supported by high-quality evidence, including Level I-II clinical trials,^16^ FDA-approved indications,^17^ and established consensus guidelines from major medical organizations.^18^^,^^19^ This selective approach resulted in an initial KG comprising ∼1.5 million core concepts and 3 million primary relationship types, focused on key clinical domains such as oncology, cardiology, infectious diseases, and endocrinology, to ensure broad relevance across major areas of patient care.

While established ontologies like UMLS and SNOMED CT provide comprehensive medical terminologies, we chose not to start with existing ontological concepts for 2 primary reasons. First, existing ontologies lack the dynamic clinical trial relationships and real-time treatment outcomes that are central to our platform’s objectives, focusing instead on static terminological standardization rather than evolving trial-specific relationships.^2^^,^^20^ Second, building upon existing ontologies would require significant preprocessing overhead to extract and restructure relevant concepts for our real-time update requirements and specific analytical focus.^21^ Furthermore, the medical domain’s inherent characteristics make a focused, iterative KG construction more practical than attempting to predefine or adapt a universal structure. Our approach therefore emphasizes building a foundational KG from high-quality, relevant sources and then dynamically enriching it.

Despite not using them as a starting structural blueprint, our system does leverage UMLS and SNOMED CT extensively for standardization purposes during the entity normalization phase, ensuring compatibility with established medical vocabularies while maintaining the flexibility needed for dynamic clinical trial analytics.^22^^,^^23^

Unlike previous methods such as GraphRAG,^24^ which dynamically construct the KG on-the-fly and require frequent LLM invocations, our system makes significant advancements by decoupling KG topology discovery from continuous KG updates. The initial phase of topology discovery relies heavily on authoritative medical guidelines and textbooks to extract a significant portion of medical entities (nodes).

Specifically, we processed 15 major clinical guidelines including NCCN Oncology Guidelines,^25^ American Heart Association Clinical Practice Guidelines,^26^ FDA Prescribing Information databases,^27^ etc We also leverage the popular medical textbooks,^28^^,^^29^ and specialty-specific references covering oncology, cardiology, and infectious diseases. In total, this initialization phase comprised around 300 documents.

This approach reduced LLM invocation costs by approximately 70% compared to on-the-fly extraction methods. Specifically, topology discovery required ∼2400 LLM calls for the initial setup based on around 300 curated documents. In contrast, a fully dynamic construction over the entire 110k document corpus would have required an estimated 8000+ calls, as it would forgo batch processing and reuse of high-confidence entities extracted during initialization.

These trusted sources provide well-structured information, allowing an estimated 80%-90% of the nodes in the KG to be established upfront. This approach minimizes the need for frequent LLM invocations in later updates. After the initial topology is set, updates to the KG are focused on efficiently adding new relationships (edges) and attributes, such as outcomes or trial results, to the existing nodes. In most cases, new nodes are only introduced when entirely novel concepts emerge, such as a breakthrough drug, with the majority of updates (∼80%) aimed at enriching existing relationships and attributes.

### Two-phase KG update strategy

Updating the KG with continuous clinical data requires an optimized process that leverages the initial KG topology while efficiently processing both structured and unstructured data. ClinicalMind proposes a 2-phase strategy designed to maximize performance, reduce redundancy, and ensure that the KG remains current with minimal computational cost.

#### Phase 1: incremental information extraction

In the first phase, we extract incremental information from structured (eg, EMRs) and unstructured data (eg, clinical trial reports, medical notes, imaging data). Since the initial KG topology already contains 80%-90% of core medical concepts (nodes), this phase focuses on discovering new relationships (edges) and enriching existing nodes with additional attributes.

For structured data sources such as EMRs, schema alignment and field mapping techniques are used to extract new relationships and clinical attributes. We implemented an automated schema alignment system that maps EMR fields to KG entities using a cascade of strategies: first, exact string matching of field names to KG entity types or attributes; second, semantic similarity scoring between field names/descriptions and KG concepts using Bio-ClinicalBERT embeddings; and third, leveraging existing medical ontology mappings (eg, LOINC to SNOMED CT via UMLS) for standardized fields like lab tests. Discrepancies or low-confidence mappings are flagged for LLM review or manual confirmation. When multiple comorbidities are documented in a single encounter, we use timestamp analysis and clinical logic rules to determine causality and precedence. For example, if diabetes and hypertension are both documented, but antihypertensive medications are prescribed before diabetes medications, this temporal sequence is captured in the relationship properties.

Furthermore, to attribute labs or medications to one of several concurrently documented comorbidities where direct temporal precedence is insufficient (eg, attributing a general lab result like C-Reactive Protein to one of multiple active inflammatory conditions), our “clinical logic rules” involve a hierarchical approach:

1. **Direct KG Knowledge Retrieval:** The system first queries the foundational KG for established, strong, and specific associations (eg, “Metformin *treats* Diabetes Mellitus Type 2,” “Elevated Serum Creatinine *is an indicator of* Acute Kidney Injury”). If a lab result or medication has a highly specific and singular link to one of the active comorbidities in the KG, this direct association is prioritized.
2. **LLM-based Contextual Disambiguation:** For ambiguous situations where a lab or medication could plausibly relate to multiple active comorbidities, or where no strong direct link exists in the KG, we leverage an LLM. The LLM is provided with a prompt containing: (1) the list of concurrently active comorbidities for the patient in that encounter, (2) the specific lab results and/or medications in question, (3) relevant contextual snippets from the EMR notes for that encounter, and (4) key elements of the patient’s relevant medical history from the KG. The prompt is designed to ask the LLM to infer the most probable primary association, for example: “Given active comorbidities (Acute Kidney Injury, Pneumonia) and lab results (Elevated Serum Creatinine, Elevated White Blood Cell Count), which comorbidity is the elevated Serum Creatinine most likely associated with in this clinical scenario? Provide your reasoning.”
3. **Relationship Attribution and Annotation:** Based on this analysis, relationships between the specific comorbidity and the lab/medication are established in the KG. These relationships can be annotated with the method of inference, and, if applicable, a confidence score derived from the LLM or rule-based certainty, to reflect the strength of the association.

This multistep process allows for more nuanced and contextually appropriate linking of clinical data points to specific conditions within complex patient encounters.

Medical standards such as SNOMED CT, ICD-10-CM, and RxNorm are applied to normalize clinical terms and link new data to existing KG nodes. Normalization follows a 3-stage pipeline: (1) exact lexical matching after canonicalization using UMLS Lexical Variant Generation tools; (2) synonym expansion through resources such as MetaMap and QuickUMLS; and (3) LLM-assisted candidate ranking for ambiguous terms, constrained by semantic-type filters and ontology cross-checks to prevent invalid mappings.

For example, a diagnosis of “Type 2 Diabetes Mellitus” (ICD-10-CM: E11.9) is mapped via the UMLS ICD10CM_SNOMEDCT_REL table to SNOMED CT concept 44054006. Similarly, “Metformin 500 mg extended-release” is normalized to RxNorm concept 860975 (metformin hydrochloride 500 mg 24HR extended-release oral tablet), ensuring consistent representation across disparate EMR systems.

For unstructured data sources, the system uses NLP and machine learning models to efficiently extract and update the clinical KG. NER models like BioBERT^4^ and ClinicalBERT^30^ extract key medical entities, while relation extraction models such as REBEL identify relationships between entities. For complex tasks requiring deeper contextual analysis, advanced LLMs like GPT-4 are used selectively. For instance, to resolve ambiguity for a term like “discharge,” which could refer to a physiological secretion or hospital discharge, the LLM is provided with the surrounding sentence or clinical note snippet. A prompt such as “Given the context: ‘…patient noted a clear nasal discharge…’, does ‘discharge’ refer to a bodily fluid or the process of leaving a healthcare facility?” allows the LLM to select the correct semantic meaning, enabling accurate mapping to the appropriate KG concept or relationship.

#### Phase 2: applying incremental updates to the KG

In the second phase, extracted relationships, attributes, and occasionally new entities from Phase 1 are integrated into the KG. Our edge insertion algorithm follows a structured process: entity verification, relationship validation against predefined ontology rules, conflict detection to identify contradictions (eg, a drug cannot both “treat” and “contraindicate” the same condition), confidence scoring based on source authority, and temporal tagging for validity periods.

The KG update process utilizes Neo4j’s MERGE operations for atomic updates, ensuring data consistency during concurrent access. Update validation employs both syntactic checks (data type validation) and semantic checks (medical logic validation using clinical decision rules).

To ensure integrity, the system conducts thorough consistency checks before applying updates. Conflicting relationships are flagged for review, and relationships extracted from unstructured text are cross-verified with structured clinical data to maintain accurate information across multiple modalities. Our resolution strategy involves presenting the conflicting statements and their sources to an LLM (GPT-4) with a prompt to identify the most likely correct assertion or to suggest a nuanced reconciliation (eg, “Drug X treats Condition Y in general, but is contraindicated in this patient due to Comorbidity Z”), and prioritizing information from structured EMR data or high-authority guidelines over inferences from unstructured notes.

## Results

To thoroughly assess the performance of the ClinicalMind platform, we conducted a series of experiments focusing on 3 key aspects: dataset scalability, hardware platform efficiency, and comparison with state-of-the-art methods. Our results demonstrate the platform’s capability to process over 110 000 clinical documents in real-time, showcasing significant improvements in speed, throughput, and scalability.

### Experiment setup

#### Platform

The evaluation was performed on a high-performance computing platform tailored for large-scale KG operations. The platform utilized 5 instances of Standard E64ads v5, each equipped with 64 vCPUs and 512 GiB of RAM, optimized for memory-intensive tasks. These instances were designed to handle the large-scale data processing required for managing and querying the clinical KG stored in Neo4j.

Additionally, the platform employed 2 instances of Standard NC24ads A100 v4, each featuring 24 cores, 220 GiB of RAM, and 64 GB of disk storage. These instances were equipped with NVIDIA A100 Tensor Core GPUs, providing the necessary hardware acceleration for tasks such as NLP processing and graph traversal. The GPU-accelerated instances significantly enhanced the system’s ability to process large-scale clinical documents and efficiently update the KG in real-time.

This configuration enabled the platform to distribute computational tasks across multiple nodes for parallel processing, optimizing both NLP and graph-based query execution. By leveraging the GPU-accelerated instances and Neo4j’s robust graph database architecture, the system achieved high throughput and low latency in handling clinical data and performing KG updates, meeting the demands of real-time clinical analytics.

#### Dataset

For our evaluation, we employed a combination of publicly available and proprietary datasets spanning both structured and unstructured data sources. The datasets include:

1. MIMIC-III Clinical Database: Health records from over 60 000 ICU patients, providing structured data for analyzing clinical treatments and outcomes.^31^
2. PubMed Central Open Access: Over 100 000 of clinical trial articles and research papers, offering unstructured data for evaluating document processing and information extraction.^32^ They were selected from 2010 to present, focusing on recent clinical trial publications.
3. Proprietary Clinical Trials: Over 10,000 clinical trial reports from pharmaceutical companies, consisting of both structured and unstructured data, used to assess ClinicalMind’s performance in handling diverse data formats. These reports cover studies conducted between 2005 and 2023, capturing nearly 2 decades of trial-level data.

Recognizing the temporal disparities, particularly with MIMIC-III (2001-2012), and the evolution of medical knowledge, our platform incorporates several mechanisms to manage temporal consistency. First, all extracted relationships and entities in the KG are, where possible, timestamped with their source document’s publication date or the EMR encounter date. Second, during KG updates, our conflict detection and confidence scoring mechanisms consider the recency and authority of the information source. For example, a treatment guideline from 2023 would supersede a conflicting statement from a 2010 textbook or an older EMR record for establishing current best practices. Third, the LLM, when used for validation or complex relationship extraction, is often provided with temporal context (eg, “based on guidelines published after 2020…”) to guide its reasoning. While perfect temporal alignment across all historical and contemporary data is challenging, these measures help ClinicalMind to prioritize current knowledge and track the evolution of clinical understanding within the KG.

Additionally, the combination of structured data (eg, EMRs) and unstructured documents (eg, clinical trial papers) enables us to evaluate the ability of ClinicalMind to handle large volumes of diverse data in both real-time and batch processing scenarios.

#### Metrics

The performance of ClinicalMind was evaluated based on 3 key metrics: latency, throughput, and scalability. Additionally, we compared its performance against several existing state-of-the-art methods for clinical document processing and KG construction. To address the critical need for clinical accuracy validation, we implemented both computational metrics and expert clinical evaluation protocols.

### Evaluation results

#### KG initialization

The initialization of the clinical KG was evaluated by processing 300 documents, consisting of medical textbooks, selected clinical trial reports, and official clinical guidance. These documents were parsed, and clinical entities such as drugs, diseases, symptoms, and patient outcomes were extracted to construct the KG. The initial processing constructed ∼1.5 million nodes and 3 million edges representing relationships between these entities (eg, “treats,” “causes,” “results in”). The final size of the KG after initialization was ∼12 GB.

Due to the complexity of entity extraction and relationship mapping, the initialization process took around 4 hours. This delay includes the time for parsing unstructured text, applying NER and RE models, and constructing the graph in the database. The graph creation and mapping of relationships between nodes are the most computationally intensive parts of the initialization process, which are further optimized by GPU acceleration during NLP tasks.

Although this initial processing time might seem long, it is a one-time cost for constructing the foundational KG from scratch. Subsequent real-time updates and queries on the KG are handled much more efficiently.

#### KG update

To evaluate the system’s capability to handle real-time updates to the KG, we processed over 110 000 clinical documents and 60 000 patient EMRs. These updates primarily involved adding new relationships (edges) and modifying attributes related to clinical trials, patient outcomes, drug efficacy, and side effects. Since clinical trial updates and patient records frequently involve updating existing information (such as dosage adjustments, side effects, and demographic data), the majority of updates were focused on edge insertions and attribute modifications rather than adding new nodes.

Across this update process, ∼800 000 new edges were added and 1.5 million attribute modifications were made. Due to the nature of clinical data and EMRs, the number of new nodes introduced to the KG was relatively low, with about 20 000 new nodes (representing new drugs, trials, or newly discovered clinical conditions) being added.

The system maintained an average update delay of 30 seconds per document or EMR. This delay includes the time to extract relevant information from each document, apply NLP for entity and relationship extraction, and integrate the extracted data into the KG.

After processing these updates, the final KG grew to ∼1.52 million nodes and 3.8 million edges. Additionally, the KG included about 5 million attributes, representing detailed clinical metadata like trial outcomes, patient demographics, and dosage information. The overall storage size of the KG reached 16 GB, demonstrating the system’s ability to handle large-scale, real-time clinical updates while maintaining efficient storage and processing capabilities.

The system is optimized for frequent edge insertions and attribute modifications, which reduces the computational overhead for real-time updates. This efficiency is particularly beneficial, as the majority of real-time updates to the KG (eg, adding new trial results or updating patient data) are edge and attribute modifications rather than node insertions. The system’s ability to process updates at an average delay of 30 seconds per data point ensures that the KG remains up-to-date with the latest clinical information in near-real-time.

#### KG queries

For evaluating the query performance, we collected a set of 100 real-world clinical questions and submitted them to the system to assess both the query response time and the accuracy of the generated results. The clinical questions included tasks such as retrieving drug-disease interactions, identifying clinical trial outcomes, and querying patient response rates. Specifically, the questions were designed to elicit a range of response formats, including factual recall (eg, specific drug names, dosage ranges—often phrases or short sentences), summarization of trial outcomes (longer sentences or short paragraphs), and identification of relationships (eg, listing associated adverse events—typically lists or phrases).

The system demonstrated an average query response time of 1.7 seconds to generate the first token of the query result. This near-instantaneous feedback was possible due to the implementation of query optimization techniques and the use of GPUs for efficient graph traversal and LLM integration, enabling faster processing.

**Figure 1.**
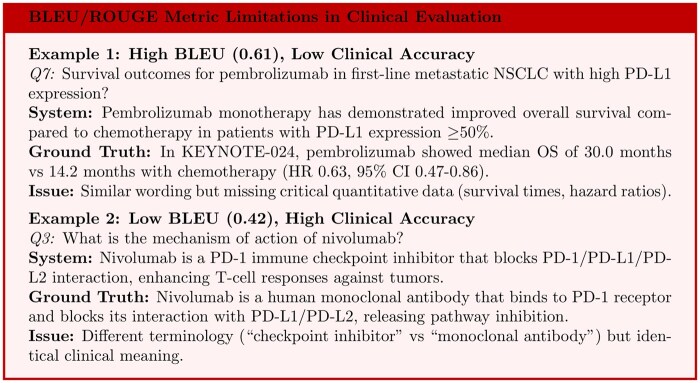
BLEU/ROUGE limitations in clinical AI: high similarity can mask incomplete answers (Example 1), while low similarity can penalize accurate responses using different medical terminology (Example 2).

In terms of computational accuracy assessment, the system’s results were initially evaluated against ground-truth answers using established text evaluation metrics such as BLEU^33^ and ROUGE^34^ scores. The system achieved an average BLEU score of 0.85 and a ROUGE score of 0.92. However, our analysis revealed significant limitations in applying these metrics to clinical evaluation contexts (Figure 1). These scores primarily measure textual similarity rather than clinical accuracy, with instances where high text similarity corresponded to clinically incomplete answers, and conversely, where clinically accurate responses using different medical terminology received lower similarity scores. Given these limitations, we recognized the need for more appropriate clinical evaluation methods.

### Expert clinical evaluation

To provide robust validation of clinical accuracy and utility, we conducted a comprehensive expert evaluation study. Clinical specialists with extensive experience in their respective domains independently assessed the quality and clinical appropriateness of system responses across multiple dimensions.

The evaluation protocol focused on 4 key aspects of clinical quality: the factual correctness of medical information provided, the completeness of essential clinical details such as dosages and contraindications, the appropriate identification of safety considerations and adverse effects, and the overall utility and actionability of responses for clinical decision-making scenarios. Each response was systematically scored using a structured 5-point assessment scale, ranging from poor to excellent clinical quality. To ensure consistency in evaluation standards, we implemented calibration procedures and assessed inter-rater agreement among evaluators. The evaluation process was designed to minimize bias through blinded assessment protocols and standardized scoring criteria.

The results demonstrated strong clinical performance across all evaluation dimensions. Overall, 89% of system responses achieved clinically acceptable quality standards, with 67% receiving ratings of very good or excellent. The assessment revealed particularly strong performance in clinical accuracy, with mean scores consistently above 4.0 on the 5-point scale. Completeness and safety assessments also showed robust performance, indicating that the system appropriately includes essential clinical information and safety considerations.

Error analysis of the responses that received lower ratings revealed specific areas for improvement. The primary issues identified included missing quantitative precision in ∼4% of responses, where specific dosages or survival statistics were omitted. An additional 4% of responses showed incomplete safety information, failing to mention important contraindications or monitoring requirements. Finally, 3% of responses provided overly general guidance that lacked actionable clinical advice. Importantly, no responses contained factually incorrect medical information, demonstrating the safety and reliability of the system’s clinical outputs.

Performance analysis across different clinical domains showed some variation in system effectiveness. Oncology-related queries achieved the highest accuracy scores, reflecting the comprehensive cancer-related content in the training dataset. However, queries related to rare diseases showed relatively lower performance, due to the limited representation of specialized literature in these areas.

The expert clinical evaluation provides essential validation that ClinicalMind achieves reliable clinical performance for the majority of queries, with particular strength in well-represented medical domains. This systematic assessment by clinical professionals offers robust evidence of the system’s practical utility in real-world clinical scenarios, addressing the recognized limitations of text-similarity metrics for healthcare AI evaluation.

## Discussion

### Key findings and contributions

Our results demonstrate significant improvements over existing methods in several key areas:

1. Efficient KG construction through separation of topology discovery and updates
2. Real-time performance for large-scale clinical document analysis
3. Clinically validated accuracy with expert evaluation achieving 89% of responses rated as “Good” or higher

### Clinical validation and practical implications

The expert evaluation provides crucial validation of ClinicalMind’s clinical utility beyond computational performance metrics. With 89% of responses achieving clinically acceptable ratings and 67% rated as “Very Good” or “Excellent,” the system demonstrates practical applicability for clinical decision support. The absence of factually incorrect medical information in expert-evaluated responses indicates robust safety mechanisms, a critical requirement for healthcare AI systems.

The domain-specific performance variations observed across different clinical areas reflect the heterogeneous representation of medical knowledge in clinical literature. Oncology-related queries demonstrated particularly strong performance, while queries involving rare diseases showed relatively lower accuracy scores. This finding highlights the importance of comprehensive training data coverage and suggests targeted enhancement strategies for underrepresented clinical domains.

### Comparison with existing approaches

The use of AI in clinical document analysis has evolved significantly. Early NLP systems like MedLEE^35^ and MetaMap^36^ focused on mapping clinical terms to standardized vocabularies. More recent transformer-based architectures like ClinicalBERT^30^ and BioBERT^4^ have improved domain-specific language understanding, outperforming traditional methods in entity recognition and relation extraction. SpaCy and Stanford CoreNLP have further extended these capabilities with specialized medical pipelines.

Deep learning approaches have broadened the scope of clinical analysis. Notably, CNNs and RNNs have advanced patient outcome prediction,^37^ while stacked autoencoders have improved disease prediction accuracy.^38^ Reinforcement learning has shown promise in optimizing treatment strategies through dynamic feedback control.^39^ In the domain of EHR analysis, RETAIN^40^ demonstrated the value of neural attention mechanisms, while Graph Convolutional Transformers^41^ have enhanced EHR structure learning.

The evolution of clinical knowledge representation spans from early terminological frameworks like SNOMED CT^8^ and RxNorm^42^ to comprehensive KG systems. Recent platforms like MIMIC-III^31^ have enabled advanced patient care analysis, while Bio2RDF^43^ and OpenBioLink^44^ have established large-scale biomedical KGs. Clinical KGs have further evolved to model complex interactions between drugs, diseases, symptoms, and outcomes,^2^ with systems like DeepPatient^38^ leveraging these structures for predictive analytics. Modern graph databases like Neo4j and AWS Neptune^1^ have made real-time clinical queries feasible.

While these approaches have advanced clinical data representation and analysis, ClinicalMind distinguishes itself through its focus on efficient real-time querying and large-scale document analysis. Our system complements existing work by providing an efficient RAG solution that could potentially integrate with and benefit from these established methods, while maintaining its emphasis on performance and scalability.

## Conclusion

This paper presents ClinicalMind that autonomously constructs a clinical trial-specific KG by parsing both unstructured and structured data. The system leverages a combination of machine learning techniques, including NLP for entity and relation extraction, and graph databases for efficient storage and querying. Our experiments demonstrated the system’s capability to handle large-scale data processing, and it can answer clinical questions in 1.7 seconds with high accuracy. Expert clinical evaluation validates the system’s practical utility, with 89% of responses achieving clinically acceptable standards, demonstrating both computational efficiency and clinical safety for real-world healthcare applications.

## Data Availability

The data that support the findings of this study are available from the corresponding author, Rui Li, upon reasonable request.
